# Supplementary material for: Heavy metal content and microbial characteristics of soil plant system in Dabaoshan mining area, Guangdong Province
Source: PLoS One. 2023 Jun 9;18(6):e0285425. doi: 10.1371/journal.pone.0285425 (PMC10256142; doi:10.1371/journal.pone.0285425)
Supplement: Attachment — Submitted filename: PONE-D-22-31232.pdf [file pone.0285425.s002.pdf]

PLOS ONE

Microbial characteristics of soil-Miscanthus floridulus plant system in Dabaoshan Lead-zinc mining area, northern Guangdong Province

--Manuscript Draft--

**Manuscript Number:**	PONE-D-22-31232
**Article Type:**	Research Article
**Full Title:**	Microbial characteristics of soil-Miscanthus floridulus plant system in Dabaoshan Lead-zinc mining area, northern Guangdong Province
**Short Title:**	Microbial characteristics of soil-Miscanthus floridulus plant system in Dabaoshan Lead-zinc mining area
**Corresponding Author:**	Jianqiao QIN, Ph.D.<br>Zhaoqing University<br>Zhaoqing, CHINA
**Keywords:**	Pb/Zn mine; Soil ecosystem; Heavy metal; Soil microbial characteristics
**Abstract:**	The disordered mining of Dabaoshan lead-zinc mineral resources in Shaoguan has brought serious harm to the regional ecological environment. The distribution of heavy metals in the soil, the activity of soil microorganisms and the accumulation characteristics of heavy metals in the dominant plant Miscanthus floridulus were studied. The results indicated that metal element contents of Miscanthus floridulus in sequence were: Zn>Pb>Cu> Cd. This study demonstrated that the elemental content of the Miscanthus floridulus plant showed Zn>Pb>Cu>Cd, with Zn being the most significantly correlated with soil elements, followed by Pb. Compared with the control group, the Miscanthus floridulus-soil system possessed obviously different soil microbial features: intensiver in microbial basal respiration strength, and higher microbial eco-physiological parameters Cmic/Corg and qCO2, but lower in soil microbial biomass. The results showed the soil enzymatic activities decreased significantly with increase of contamination of heavy metals, especially dehydrogenase and urease activities. With the increase of the content of heavy metals in the mining area soil, the intensity of soil biochemical action in the mining area (Q1, Q2) soil decreased significantly, and the biochemical action showed a significant negative correlation with the content of heavy metals in the soil. Compared with the non mining area (Q8) soil, the intensity of soil ammonification, nitrification, N fixation and cellulose decomposition decreased by 43.2%~71.1%, 70.1%~92.1%, 58.7%~87.8% and 55.3%~79.8% respectively. The decrease of soil microbial activity weakened the circulation rate and energy flow of C and N nutrients in the soil of the mining area.
**Order of Authors:**	Jianqiao QIN, Ph.D.
	Zhiqiang Yan
	Huarong Zhao
	Peng Zhao
	Yibo Yao
	Xi Chen
**Additional Information:**	
**Question**	**Response**
**Financial Disclosure**<br><br>Enter a financial disclosure statement that describes the sources of funding for the work included in this submission. Review the submission guidelines for detailed requirements. View published research	Special Project of Key Areas of Colleges<br>and Universities in Guangdong Province (Science and Technology Promoting Rural Revitalization)<br>“Research and Development of Key Technologies for Resource Utilization of Manure from Large-<br>Scale Livestock and Poultry Breeding in Rural Areas of Western Guangdong”<br>(No.:2021ZDZX4023)

Powered by Editorial Manager® and ProduXion Manager® from Aries Systems Corporation

articles from *PLOS ONE* for specific examples.<br><br>This statement is required for submission and **will appear in the published article** if the submission is accepted. Please make sure it is accurate.<br><br>**Unfunded studies**<br>Enter: *The author(s) received no specific funding for this work.*<br><br>**Funded studies**<br>Enter a statement with the following details:<br>• Initials of the authors who received each award<br>• Grant numbers awarded to each author<br>• The full name of each funder<br>• URL of each funder website<br>• Did the sponsors or funders play any role in the study design, data collection and analysis, decision to publish, or preparation of the manuscript?<br>• **NO** - Include this sentence at the end of your statement: *The funders had no role in study design, data collection and analysis, decision to publish, or preparation of the manuscript.*<br>• **YES** - Specify the role(s) played.<br><br>* typeset	
**Competing Interests**<br><br>Use the instructions below to enter a competing interest statement for this submission. On behalf of all authors, disclose any competing interests that could be perceived to bias this work—acknowledging all financial support and any other relevant financial or non-financial competing interests.<br><br>This statement is required for submission and **will appear in the published article** if the submission is accepted. Please make sure it is accurate and that any funding sources listed in your Funding Information later in the submission form are also declared in your Financial Disclosure statement.	The authors have declared that no competing interests exist.

View published research articles from [*PLOS ONE*](#) for specific examples.<br><br>**NO authors have competing interests**<br><br>Enter: *The authors have declared that no competing interests exist.*<br><br>**Authors with competing interests**<br><br>Enter competing interest details beginning with this statement:<br><br>*I have read the journal's policy and the authors of this manuscript have the following competing interests: [insert competing interests here]*<br><br>* typeset	
**Ethics Statement**<br><br>Enter an ethics statement for this submission. This statement is required if the study involved:<br><br>• Human participants<br>• Human specimens or tissue<br>• Vertebrate animals or cephalopods<br>• Vertebrate embryos or tissues<br>• Field research<br><br>Write "N/A" if the submission does not require an ethics statement.<br><br>General guidance is provided below. Consult the submission guidelines for detailed instructions. **Make sure that all information entered here is included in the Methods section of the manuscript.**	"N/A"

**Format for specific study types**<br><br>**Human Subject Research (involving human participants and/or tissue)**<br>• Give the name of the institutional review board or ethics committee that approved the study<br>• Include the approval number and/or a statement indicating approval of this research<br>• Indicate the form of consent obtained (written/oral) or the reason that consent was not obtained (e.g. the data were analyzed anonymously)<br><br>**Animal Research (involving vertebrate animals, embryos or tissues)**<br>• Provide the name of the Institutional Animal Care and Use Committee (IACUC) or other relevant ethics board that reviewed the study protocol, and indicate whether they approved this research or granted a formal waiver of ethical approval<br>• Include an approval number if one was obtained<br>• If the study involved *non-human primates*, add *additional details* about animal welfare and steps taken to ameliorate suffering<br>• If anesthesia, euthanasia, or any kind of animal sacrifice is part of the study, include briefly which substances and/or methods were applied<br><br>**Field Research**<br><br>Include the following details if this study involves the collection of plant, animal, or other materials from a natural setting:<br>• Field permit number<br>• Name of the institution or relevant body that granted permission	
**Data Availability**<br><br>Authors are required to make all data underlying the findings described fully available, without restriction, and from the time of publication. PLOS allows rare exceptions to address legal and ethical concerns. See the PLOS Data Policy and FAQ for detailed information.	Yes - all data are fully available without restriction

A Data Availability Statement describing where the data can be found is required at submission. Your answers to this question constitute the Data Availability Statement and **will be published in the article**, if accepted.<br><br>**Important:** Stating 'data available on request from the author' is not sufficient. If your data are only available upon request, select 'No' for the first question and explain your exceptional situation in the text box.<br><br>Do the authors confirm that all data underlying the findings described in their manuscript are fully available without restriction?	
**Describe where the data may be found in full sentences. If you are copying our sample text, replace any instances of XXX with the appropriate details.**<br><br>• If the data are **held or will be held in a public repository**, include URLs, accession numbers or DOIs. If this information will only be available after acceptance, indicate this by ticking the box below. For example: *All XXX files are available from the XXX database (accession number(s) XXX, XXX.).*<br>• If the data are all contained **within the manuscript and/or Supporting Information files**, enter the following: *All relevant data are within the manuscript and its Supporting Information files.*<br>• If neither of these applies but you are able to provide **details of access elsewhere**, with or without limitations, please do so. For example:<br><br>*Data cannot be shared publicly because of [XXX]. Data are available from the XXX Institutional Data Access / Ethics Committee (contact via XXX) for researchers who meet the criteria for access to confidential data.*<br><br>*The data underlying the results presented in the study are available from (include the name of the third party*	All relevant data are within the manuscript and its Supporting Information files.

and contact information or URL).<br>• This text is appropriate if the data are owned by a third party and authors do not have permission to share the data.<br><br>* typeset	
Additional data availability information:	

Manuscript

Click here to access/download;Manuscript;paper Jianqiao Qin.doc

Microbial characteristics of soil-*Miscanthus floridulus* plant system in Dabaoshan Lead-zinc mining area, northern Guangdong Province

Jianqiao Qin[1]*, Zhiqiang Yan[2], Huarong Zhao[3]*, Peng Zhao[4], Yibo Yao[5], Xi Chen[6]

(1 Guangdong Provincial Key Laboratory of Environmental Health and Land Resource, School of Environmental and Chemical Engineering, Zhaoqing University, Zhaoqing, 526061, China; 2 Shunde Polytechnic, Shunde 528300, China; 3 School of Environmental Science and Engineering, Guilin University of Technology, Guilin 541004, China; 4 South China Institute of Environmental Sciences, Ministry of Ecology and Environment of the People's Republic of China, Guangzhou, 510655, China; 5 Guangdong Provincial Academy of Environmental Science，Guangzhou, 510045, China; 6 School of Environmental Science and Engineering, Sun Yat-Sen University, Guangzhou, Guangdong 510275, China)

* Correspondence: qinjianqiaosci@126.com (J.Q.); zhaohuar@mail3.sysu.edu.cn (H.Z.)

Abstract: The disordered mining of Dabaoshan lead-zinc mineral resources in Shaoguan has brought serious harm to the regional ecological environment. The distribution of heavy metals in the soil, the activity of soil microorganisms and the accumulation characteristics of heavy metals in the dominant plant *Miscanthus floridulus* were studied. The results indicated that metal element contents of *Miscanthus floridulus* in sequence were: Zn>Pb>Cu> Cd. This study demonstrated that the elemental content of the *Miscanthus floridulus* plant showed Zn>Pb>Cu>Cd, with Zn being the most significantly correlated with soil elements, followed by Pb. Compared with the control group, the *Miscanthus floridulus*-soil system possessed obviously different soil microbial features: intensiver in microbial basal respiration strength, and higher microbial eco-physiological parameters Cmic/Corg and qCO_2, but lower in soil microbial biomass. The results showed the soil enzymatic activities decreased significantly with increase of ~~contamination of heavy metals~~, especially dehydrogenase and urease activities. With the increase of the content of heavy metals in the mining area soil, the intensity of soil biochemical action in the mining area (Q1, Q2) soil decreased significantly, and the biochemical action showed a significant negative correlation with the content of heavy metals in the soil. Compared with the non mining area (Q8) soil, the intensity of soil ammonification, nitrification, N fixation and cellulose decomposition decreased by 43.2%~71.1%, 70.1%~92.1%, 58.7%~87.8% and 55.3%~79.8% ~~respectively.~~ The decrease of soil microbial activity weakened the circulation rate and energy flow of C and N nutrients in the soil of the mining area.

Key words: Pb/Zn mine; Soil ecosystem; Heavy metal; Soil microbial characteristics

1. Introduction

The dense distribution of non-ferrous metal mines in the south of China has resulted in a large number of soils being damaged by mining or contaminated with heavy metals, and has caused serious damage to soil ecosystems [1,2] . Soil contamination in mining areas can also be transported by surface runoff and biogeochemistry [3-5], endangering the environmental quality of adjacent areas, while contaminated agricultural products can endanger human health through

the food chain [6,7]. Therefore, the reclamation of mine land to ~~restore and~~ reuse degraded land has become one of the hot issues in ~~environmental science and soil science~~ [1]. At present, there are few investigations on soil reclamation in non-ferrous metal mines in China, and few studies have been reported on the relationship between soil microbial activity and soil heavy metal content and plant heavy metal accumulation in mining areas[8]. Due to the long-term existence of heavy metal pollution, the microbiota of soil in mining areas had changed significantly, with different levels of sensitivity to heavy metal pollution and significant changes in the biochemical characteristics of soil microorganisms, while some plants had gradually evolved into heavy metal accumulation ecotypes through long-term evolution and natural selection, which had an important role in the reclamation of mining areas [9,10]. Therefore, actively carrying out research in this area to clarify the response mechanism between heavy metal accumulation of plants and soil microbiological and biochemical characteristics in mining areas was important for understanding the nature and causes of soil ecosystem degradation in mines [11-13], and also provides a scientific basis for the search of heavy metal hyperaccumulating plants, which was important for promoting the restoration and reconstruction of mine soil ecosystems[14].

This paper studies the distribution, microbial activity, soil enzyme activity and plant heavy metal accumulation characteristics of the soil ecosystem in Dabaoshan lead-zinc mining area, a typical mining area of mineral resources in northern Guangdong, to provide biological basis for the assessment of contaminated soil environmental quality in similar mining areas and the search for super accumulated plant germplasm resources, The purpose is to provide a theoretical basis for the rational use of soil and the phytoremediation of contaminated soil in this area.

2. Materials and Methods

2.1 Overview of the Study Area

The Dabaoshan Mine is located at the junction of Qujiang District and Wengyuan County in Shaoguan City, Guangdong Province, with a watershed influence of 113°40′-113°43′ E and 24°30′-24°36′N. The region is located in a warm, humid and rainy subtropical monsoon climate with strong weathering of the surface rocks[15,16]. The Dabaoshan Mine is a large iron-polymetallic sulphide associated deposit with a limonite ore in the upper part of the main ore and a copper-sulphide ore in the lower part, with associated non-ferrous ores such as tungsten, bismuth, molybdenum, gold and silver [17-21] (Fig. 1). Beneficiation tailings and waste

rock are mainly discharged in two large tailings impoundments (Tielong tailings impoundment and trough-to-pit tailings impoundment) formed by tailings dams and partially flooded throughout the year[22]. The tailings and waste rock contain large amounts of various metal sulphides such as pyrite and chalcopyrite, which are quickly oxidized and form acidic effluent when in contact with the air as a result of various mining activities, while releasing large amounts of toxic and harmful heavy metal ions[23]. The acidic wastewater carrying toxic heavy metal ions was discharged from the two tailings ponds and flows downstream along the river valley to the confluence at Liangqiao and then into the Heng Shi River, where it was joined by water from Chen Gong Wan (a non-mining polluted area) in the middle and lower reaches and eventually flows downstream into the river[24,25]. The acidic wastewater from the tailings pond, which contains a large amount of toxic heavy metal ions, was discharged directly into the river downstream without treatment, causing a serious deterioration of the ecological environment along the mine and its watershed. The most serious problem was the village of Shangba in Xinjiang Town, which was located downstream, where the river water was red in colour, the bottom mud was black, the fish and shrimps in the river are extinct, and the farmland irrigated by the river water has been damaged by the soil quality, and the crop yield was decreasing year by year, and the severely deteriorated ecological environment had also damaged the health of the villagers [26-30].

Fig.1 Location of Dabaoshan mining area and sampling points

2.2 Spot sampling and sample processing

The study region includes the mine waste land, the tailings pond and the Heng Shi River affected by mine wastewater, and the sewage irrigation area downstream to Shangba village. The vegetation in the sample plot was mainly grassland, and the Miscanthus floridulus community was dominant, accounting for more than 70%, with 60% ~ 80% coverage. The vegetation was flourishing and showed cluster growth. Accordingly, eight sampling points were set up (Fig. 1), namely, the abandoned land at the top of the Dabaoshan mine [Q1], the hillside of the mine [Q2], the trough-to-pit tailings reservoir [Q3], the downstream tributary of the Tielong tailings reservoir [Q4] (5 km from the mine extraction site), adjacent to the irrigation canal in Shangba Village [Q5] (12 km from the mine extraction site), near the farmland in Xiaba

Village [Q6] (16 km from the mine extraction site), the Suoyikeng [Q7] (non-mining contaminated area), Hewu Village [Q8] (non-mining contaminated area). A 1 m x 1 m sample square was set up in different sections for community survey, including plant species, population density, height and cover of *Miscanthus floridulus*.

Table 1　Growth and distribution characteristics of the *M. floridulus* population in Dabaoshan mining area

Mining area	Geographical location	Distance from the main mining area /km	Plant height /cm	Density /（plant·m^{-2}）	Coverage /%
Q1	N24°33′36.6″E113°43′14.0″	1	210±25.25	36	80～90
Q2	N24°34′06.0″E113°43′44.3″	2	200±22.45	31	70～80
Q3	N24°33′32.1″E113°43′23.8″	3	190±18.30	25	60～70
Q4	N24°33′38.1″E113°44′16.8″	5	178±15.65	21	55～65
Q5	N24°30′14.2″E113°44′06.9″	12	165±13.95	16	50～60
Q6	N24°28′46.8″E113°47′46.1″	16	150±11.18	13	40～50
Q7	N24°29′57.1″E113°49′20.8″	25	145±13.95	11	40～45
Q8	N24°30′03.1″E113°49′29.6″	30	141±11.18	11	40～50

In addition, 5~10 *Miscanthus floridulus* plants were randomly collected in each quadrat and mixed into one sample. Soil samples were collected at a depth of 0-20 cm along an S-shaped route, placed in sterile paper bags and taken back to the laboratory as soon as possible. Part of the fresh soil was ground through a 2 mm sieve, adjusted to the appropriate moisture content and stored at ~~0-4 °C~~ for testing soil microbiological indicators; the other part of the soil was air-dried and used to determine basic soil physical and chemical properties and heavy metal content. The basic chemical properties were shown in Table 2.

Table 2　Some chemical properties of soil samples tested

Soil Sample No.	Organic matter（g·kg^{-1}）	Effective phosphorus（mg·kg^{-1}）	Nitrogen alkali decomposition（mg·kg^{-1}）	pH
Q1	14.649	32.255	30.1	3.15
Q2	17.235	12.033	24.5	3.16
Q3	2.725	36.743	63.7	3.48
Q4	18.012	41.233	55.6	4.04
Q5	15.451	36.740	60.5	4.48
Q6	19.112	50.212	63.1	4.71
Q7	20.652	48.568	66.4	6.55
Q8	36.682	79.653	71.5	6.82

2.3 Determination method

Soil and plant heavy metal content: samples of *Miscanthus floridulus* were divided into roots, stems and leaves, washed with deionized water, dried to constant weight at 70 °C and crushed through an 80 mesh sieve. The samples were dry ashed and fixed with hydrochloric acid and then determined by atomic absorption spectrophotometry. The total amounts of soil Zn, Pb, Cu and Cd were digested with HCl, HF and perchloric acid and determined by ICP-OES (Optima5300DV, Perkin-Elmer Instruments, USA); the effective state of the corresponding heavy metals in the soil was extracted with 0.1 mol L^{-1} HCl (liquid: soil = 5:1) and the solution to be measured was determined by ICP-OES.

The basic chemical properties of the soil were determined using the soil agrochemical analysis method [30]: soil pH was measured by a pH meter after mixing water and soil at 2.5:1; organic matter was determined using the potassium dichromate volumetric method; nitrogen alkali decomposition was determined using the alkaline diffusion method; and the effective phosphorus was measured using the molybdenum blue colourimetric method [31] after extracting the soil samples with 0.5 mol L^{-1} sodium bicarbonate.

Soil microbial biomass carbon: using the chloroform fumigation-K_2SO_4 extraction method [30,32], the organic carbon in the extract was determined using an automatic total organic carbon analyzer (Shimadzu, TOC-500), and the difference between the measured organic carbon values of the chloroform fumigated and unfumigated soil (Fc), divided by the conversion factor Kc (0.45), was obtained as the soil microbial biomass carbon content (Cmic, mg kg^{-1}), i.e.: Cmic = Fc/0.45.

Soil basal respiration: measured by closed incubation alkaline absorption titration [33], 20 g of fresh soil sample was weighed into a 500 ml culture flask and the soil was spread evenly flat on the bottom, and the soil water content was adjusted to 60% of the field holding capacity. A 25 ml flask was placed on top of the soil in the flask, then 10 ml of 1 mol L^{-1} NaOH solution was drawn into it, the flask was sealed with a cap and incubated at a constant temperature of 28 °C for 1 month, and the amount of CO_2 released was measured every 24 h. Determination of soil biochemical strength [26]: soil ammonification strength was determined by the soil incubation method, and NH_4^+-N content was determined by the semi-micro Kjeldahl distillation method; nitrification strength was determined by the solution incubation method, and NO_3^--N reduction was determined by the colorimetric method; nitrification strength was determined by the soil

incubation method, and the increase in total soil Nitrogen was analyzed; soil cellulose decomposition strength was determined by the buried slice method, and the weight loss of cloth was analyzed.

Soil enzymatic activity was determined using the literature [30]: 3,5-dinitrosalicylic acid colorimetric method for soil sucrose enzyme activity [34]; sodium phenol colorimetric method for soil urease activity [35,36]; sodium phosphate colorimetric method for soil acid phosphatase activity [37]; potassium permanganate titration method for soil catalase activity [38-40]; ninhydrin colorimetric method for soil protease activity [41]; and TTC colorimetric method for soil dehydrogenase activity [42].

2.4 Data Processing

Statistical analysis of data is performed using a combination of Microsoft Excel 2003 and SPSS 16.0 software and the significance of differences between means were analyzed using Duncan's multiple comparisons (SSR test, $p < 0.05$).

3. Results and discussion

3.1 Heavy metal content of soil-plant systems in the Dabaoshan Pb-Zn mining area

3.1.1 Analysis of heavy metal content in soils

The results of the analysis of the total and effective contents of Zn, Pb, Cu and Cd in the test soils (Table 3) show that the contents of Zn, Pb, Cu and Cd in the soils of six sampling points in the mine area were all above the secondary standard for soil environmental quality (GB15618-1995). The Zn, Pb, Cu and Cd contents of the soil in the mine area gradually decreased from the centre of the mine area to the periphery of the mine area, and the corresponding total mean values were 10. 24, 19. 98, 119.10 and 21.35 times higher than those of the control soil samples (soil samples No. 7 and 8). 47.57 times higher than the control soil samples (soil samples 7 and 8), indicating that the soils in the mining area were all contaminated with heavy metals to different degrees. The data in Table 2 showed significant differences in the total and effective amounts of Zn, Pb, Cu and Cd in each soil sample from the centre of the tailings pond to the periphery by the LSD0.05 test ($P < 0.05$). This difference may be related to the degree of mixing of tailings sand with soil in different zones under the action of external camp forces (mainly in surface runoff water erosion). The closer to the tailings pond, the higher the total tailings deposition and the correspondingly higher heavy metal content in the soil

[28,43].

Table 3 Heavy metal contents of soil samples collected in the mine area (mg·kg^{-1})

Soil Sample No.	Heavy metals full amount				Active state heavy metal content			
	Zn	Pb	Cu	Cd	Zn	Pb	Cu	Cd
Q1	1768.25	1241.13	1698.75	9.13	291.75	68.5	238.5	1.42
Q2	780.88	1000.5	1572.5	5.11	15.07	56.43	127.2	0.09
Q3	640.50	886.25	893.63	1.88	53.95	98.8	149.48	0.47
Q4	440.25	750.25	695.20	1.25	50.25	90.35	105.5	0.40
Q5	295.25	602.75	288.75	0.63	68.83	138.03	47.79	0.51
Q6	250.33	350.25	150.25	0.55	40.25	58.30	40.25	0.30
Q7	161.75	83.63	20.1	0.25	3.58	7.61	5.7	0.14
Q8	60.50	35.13	6.38	0.13	1.56	6.03	1.33	0.01
a*	250.00	200.00	50.00	0.30	-	-	-	-

Note: a*, Soil Environmental Quality Class II Standard (GB15618-1995)

3.1.2 Heavy metal element content of different parts of the *Miscanthus floridulus* plant

As shown in Table 3, the accumulation of Zn, Pb, Cu and Cd in the shoot of *M. floridulus* increased significantly with the increase of soil heavy metal content. See Table 4 for the heavy metal content in different parts of the *M. floridulus*. According to Table 4, The average contents of Zn, Pb, Cu and Cd in *M. floridulus* were as follows: Zn(110.21±18.12 mg/kg) > Pb(71.11±5.97 mg/kg) > Cu(50.15±5.37 mg/kg) > Cd(3.02±0.81 mg/kg). The content of elements in roots, stems and leaves was Zn > Pb > Cu > Cd. The content of Zn in roots was (123.25±14.10) mg/kg, which was 1.6 times that of Pb, 2.4 times that of Cu and 35.9 times that of Cd, respectively. The contents of Zn, Pb, Cu and Cd were different in different parts of the plant, showing root > stem > leaf.

Table 4 Content of elements in different parts of *M. floridulus* (mg/kg)

Soil sample No.	Zn			Pb			Cu			Cd		
	root	stem	leaf	root	stem	leaf	root	stem	leaf	root	stem	leaf
Q1	195.21	93.78	85.17	160.18	56.08	36.35	82.5	51.15	43.28	30.22	18.22	6.45
Q2	143.25	72.33	52.19	121.56	37.04	13.28	71.1	27.29	21.21	13.55	11.25	3.51
Q3	123.15	62.75	30.15	81.16	15.05	5.25	52.1	7.25	3.25	3.52	1.22	0.41
Q4	94.55	53.44	11.18	52.36	8.01	4.65	32.6	5.26	2.22	1.56	0.29	0.29
Q5	85.28	42.65	8.11	21.15	4.03	3.25	22.3	3.35	1.25	0.52	0.22	0.15
Q6	76.29	31.85	3.16	11.33	1.05	0.75	11.0	2.55	0.23	0.33	0.20	0.11
Q7	72.19	30.75	3.03	9.30	1.01	0.63	10.79	2.33	0.20	0.31	0.21	0.13
Q8	71.11	29.85	2.96	8.93	1.05	0.61	10.05	2.18	0.19	0.26	0.17	0.11

Average content	110.21±18.12	71.11±5.97	50.15±5.37	3.02±0.81

3.1.3 *M. floridulus* on the relationship between mango plants and soil heavy metal elements

The *M. floridulus* in relation to the soil elements were analyzed as shown in Table 5. The results showed that the correlation between Zn in all parts (root, stem and leaf) and soil Zn was significant ($p<0.05$), with the correlation between root and stem being highly significant ($p<0.01$); the correlation between Cu and Cd in root and soil was significant; and the correlation between Pb in stem was highly significant. It indicated that the correlation of Zn in all parts of the *M. floridulus* was better than the correlation of Pb, Cu and Cd elements, and that the uptake and accumulation of Zn by the plant increases with the increase of soil Zn content. It also shows that even the same plant did not absorb and accumulate different elements differently, reflecting the characteristics of the plant itself on the one hand, and the influence of soil elements on the plant and their ability to migrate in the plant on the other[44,45].

Table 5 Correlation coefficient between the content of elements in *M. floridulus* and that in soils

Parts of plant	Zn	Pb	Cu	Cd
Root	0.725**	0.171	0.489*	0.425*
Stem	0.621**	0.528**	0.365	0.025
Leaf	0.465*	0.321	0.320	0.116

Note: *p≤0.05, **p≤0.01, n=23

3.2 Variations in the microbiological characteristics of the Dabaoshan lead-zinc mining area

The changes in soil microbial characteristics parameters were shown in Fig. 2. It can be seen that from Q8 and Q7 (control soil samples) to Q1, as the heavy metal content increased, the more luxuriant the growth of *M. floridulus*, the higher the rate of basal microbial respiration, while the soil microbial biomass tended to decrease, and the t-test showed a significant difference between them ($p<0.05$). Fig.2 also showed that two microbial physiological and ecological parameters, the ratio of soil microbial carbon to total organic carbon (microbial quotient = Cmic/Corg) and the ratio of soil basal respiration intensity to microbial carbon

(metabolic quotient qCO_2 = Rmic/Cmic), showed similar trends, with significant increases in both parameters from Q8 to Q1 in control soils ($p<0.05$). This indicated that the metabolic capacity of microorganisms was significantly altered in the soil-plant system of the Pb-Zn mine. This may be related to the dense growth of *M. floridulus* in the above-ground part.

As seen from the results of the study, the microbial quotient (Cmic/Corg) and metabolic quotient (qCO_2) of the Pb-Zn mining soil-*M. floridulus* plant system showed an increasing trend compared to the control soil, and the soil respiration rate was significantly enhanced, which may be caused by a significant increase in the intensity of microbial activity due to the resistance of microorganisms to long-term stress by heavy metals (especially Cu) [46,47]. In contrast, soil microbial biomass showed a decreasing trend, probably due to microorganisms maintaining a low substrate utilization rate [48], indicating that the magnitude of soil microbial biomass in the Pb-Zn mining soil-*M. floridulus* system did not correspond well to the intensity of microbial physiological activity and that the metabolic capacity of microorganisms was significantly altered. In such an environment, microorganisms need to consume more energy in order to maintain their normal life activities, resulting in less efficient use of energy carbon by soil microorganisms [49].

Fig.2 Variation of microorganisms features from different sites (SMB=Soil Microbial Biomass)

Table 6 showed a comparison of the correlations between microbial parameters. Under normal conditions, microbial respiration intensity was positively correlated with microbial biomass, so the soil respiration rates of the control soil samples (Q7 and Q8) in Table 5 were positively correlated with microbial load, but this correlation became weaker in the mine site (Q6 to Q1) soils, indicating that the soil microbial load did not correspond well to its respiration intensity in the Pb-Zn mine soil- *M. floridulus* system. Similarly, the negative correlations of Cmic/Corg and qCO_2 with microbial biomass show that lower microbial loads in Pb-Zn mining soils correspond to higher microbial physiological activity[23,50]. Although Cmic/Corg was usually negatively correlated with soil organic carbon, and the organic carbon content of the mine (Q1-Q6) soils was significantly lower than that of the control soils, the negative correlation between Cmic/Corg and Corg (organic carbon) becomes increasingly poor from the control soils

(Q7 and Q8) to the mine (Q1-Q6) soils, reflecting the fact that in a soil ecosystem as specific as the Pb-Zn mine, the Cmic/Corg and Corg (organic carbon) correlations become increasingly poor. It also reflected the fact that the intensity of soil microbial physiological activity had changed significantly in such a special soil ecosystem as the Pb-Zn mine [51].

Table 6 Correlation between soil microbial characterisation parameters (n=3)

Item	Q1	Q2	Q3	Q4	Q5	Q6	Q7	Q8
Cmic/Corg & SMB	-0.825	-0.755	-0.625	-0.447	-0.208	-0.101	-0.059	-0.053
qCO_2 & SMB	-0.881	-0.813	-0.795	-0.719	-0.638	-0.311	0.072	0.075
Basic Breathing & SMB	0.475	0.619	0.664	0.773	0.820	0.882	0.901	0.911
Cmic/Corg & Corg	-0.568	-0.643	-0.755	-0.785	-0.814	-0.828	-0.939	-0.950

3.3 Analysis of soil enzyme activity in the Dabaoshan Pb-Zn mining area

The results of the analysis of the six enzyme activities of each test soil were shown in Fig. 3. As can be seen from Fig. 3, there was a certain degree of variation in enzyme activity between the soil samples, with the enzyme activity of the soil in the tailings contaminated area being lower and the control soil (Q8) having the highest values for all the enzymes, with the urease, protease and dehydrogenase activities showing greater variation, followed by phosphatase activity and less variation in sucrase and peroxidase activities. Fig.3 also showed that there was also a large variation in the magnitude of the different types of enzyme activities in the mine soils. The mean values of urease, dehydrogenase, protease, phosphatase, sucrase and catalase activities were 43.81%, 27.40%, 57.86%, 44.19%, 83.04% and 58.16% of those of the control soil respectively.

Fig.3 The enzymatic activities of soil samples tested ((A) is the Urease activity,(B) is the Sucrose enzyme activity, (C) is the Protease activity, (D) is the Phosphatase activity, (E) is the Catalase activity,and (F) is the Dehydrogenase activity.)

Note: Error bars mean standard deviation; different letters in the same group indicate significant difference at P<0. 05 according to Duncan's multiple range tests.

The results of the factor analysis of the heavy metal content and basic chemical properties of the test soils were shown in Table 7, which yielded a variance contribution of 98.60% for the

first principal factor of heavy metal element content, indicating that this principal factor can reflect the variability of the whole variable system of the test soils, which showed that the main influencing factor leading to the reduced enzyme activity of the soil in this tailings contaminated area was the heavy metal element content. This was related to the inhibitory effect of heavy metals on enzymes [19,51,54]. The mechanism of action may be due to the combination of the active site of the enzyme molecule, the sulfhydryl group, and the imidazole-containing ligand, forming a more stable complex, resulting in a competitive inhibition with the substrate, or it may be due to the inhibition of the growth and reproduction of soil microorganisms by heavy metals, reducing the synthesis and secretion of enzymes in the organism and finally leading to a decrease in soil enzyme activity[55].

Table 7 Factor analysis of heavy metal contents and chemical properties of soil

Factor	Zn	Pb	Cu	Cd	Organic matter （$g\cdot kg^{-1}$）	Effective phosphorus （$mg\cdot kg^{-1}$）	Nitrogen alkali decomposition （$mg\cdot kg^{-1}$）	pH
Contribution of variance %	69.448	16.418	11.360	1.374	1.176	0.206	0.018	0.000
Cumulative contribution of variance%	69.448	85.866	97.226	98.600	99.776	99.982	100.000	100.000

3.4 Intensity of soil biochemical action

The intensity of soil ammonification, nitrification, Nitrogen fixation and cellulose decomposition was carried out with the direct participation of all major physiological groups of soil microorganisms, and the soil played an important role in maintaining the C and N balance of its ecosystem with the coordination of these microbial communities [19]. The intensity of soil biochemical action was usually considered as one of the comprehensive indicators of soil microbial activity [28]. The results of the analysis (Table 8) showed that the intensity of soil biochemical action in the mining area (Q1 and Q2) soils was significantly reduced, compared with the non-mining area (Q7 and Q8) soils, in which the intensity of soil ammonification, nitrification, Nitrogen fixation and cellulose decomposition decreased by 43.2%~71.1%, 70. 1%~92.1%, 58.7%~87.8% and 55.3%~79.8%, which shown a significant negative correlation ($p<0.05$) with the content of heavy metals in the soil, leading to a weakened rate of nutrient cycling of C and N elements in the soil [29], thus reducing the intensity of the supply of effective

nutrients in the soil, resulting in the current situation where the above-ground part grows only with a large number of heavy metal-tolerant plants (*M. floridulus*). The mechanism of tolerance deserves further study[52,53].

Table 8 Strength of biochemical action of soil at different levels of contamination g kg^{-1}

Soil Sample No.	Ammonification	Nitrification	Nitrogen fixation	Cellulosic decomposition strength
Q1	0.104±0.013c	0.013±0.006e	0.025±0.008d	1.21±0.080f
Q2	0.125±0.014c	0.023±0.007e	0.045±0.031d	1.55±0.201e
Q3	0.147±0.020b	0.041±0.011d	0.065±0.022c	2.17±0.103d
Q4	0.197±0.020b	0.048±0.018d	0.085±0.012c	2.67±0.105d
Q5	0.215±0.079b	0.068±0.010c	0.105±0.015c	3.16±0.150c
Q6	0.255±0.010b	0.099±0.012b	0.155±0.019b	4.66±0.200b
Q7	0.348±0.020a	0.165±0.011a	0.206±0.020a	5.96±0.031a
Q8	0.358±0.020a	0.175±0.012a	0.216±0.020a	5.98±0.041a

Note: Different alphabets after the data in the same column indicate significant differences ($p < 0.05$), n = 3.

4 .Conclusions

(1) The content of heavy metal elements in the soil of lead-zinc mining area was higher than that of the control soil, and the soil of the mining area was polluted to different degrees. The heavy metal content in the dominant species of *M. floridulus* in the mining area was significantly positively correlated with the total and available content of heavy metal in the soil. From the mine contaminated area to the control area, the heavy metal content in soil and plant gradually decreased.

(2) In the soil of lead-zinc mining area, the intensity of soil microbial physiological activity has changed obviously. With the increase of soil heavy metal content, soil microbial biomass carbon decreased gradually, and soil microbial biomass carbon was significantly correlated with the total and available state of polluted heavy metals, while soil basic respiration and microbial metabolism quotient increased with the increase of soil heavy metal content. Compared with non-mining soil, the activities of several soil enzymes in mining soil were inhibited to a certain extent

(3) Decreased soil microbial activity was one of the most important signs of damage to soil ecosystems in mining areas and one of the most important factors in the ecological evolution of soil microbes in mining areas. Reduced soil microbial activity weakens the cycling rate and

energy flow of C and N nutrients in mine soils.

Author Contributions: Project administration and writing—review and editing, J.Q.; conceptualization , Z.Y. and H.Z.; formal analysis, Y.Y.; methodology, P.Z.; investigation, X.C. All authors have read and agreed to the published version of the manuscript.

Funding: This work was supported by the Guangdong Provincial Key Laboratory of Environmental Health and Land Resource (project number: 2020B121201014); Special Project of Key Areas of Colleges and Universities in Guangdong Province (Science and Technology Promoting Rural Revitalization) "Research and Development of Key Technologies for Resource Utilization of Manure from Large-Scale Livestock and Poultry Breeding in Rural Areas of Western Guangdong" (No.:2021ZDZX4023); Innovation Team Project of Colleges and Universities in Guangdong Province (2021KCXTD055).

Institutional Review Board Statement: Not applicable.

Informed Consent Statement: Not applicable.

Data Availability Statement: The data presented in this study are available on request from the corresponding author.

Conflicts of Interest: The authors declare no conflict of interest.

References

1. Teng, Y.; Huang, C.Y.; Long, J.; University, Z.; Hangzhou; Chin. Studies on soil enzymatic activities in areas contaminated by tailings from Pb,Zn,Ag mine. *China Environmental Science* **2002**.
2. Jian, L. Preliminary study on soil microbes and soil biochemical activities in mining wasteland. *Acta Ecologica Sinica* **2003**, *23*, 496-503.
3. Kim, D.-M.; Kwon, O.-H.; Oh, Y.S.; Lee, J.-S. Determination of soil contamination sources in mining area using Zn/Cd ratios with mobile Cd. *Environmental Geochemistry and Health* **2021**, *43*, 4061-4074, doi:10.1007/s10653-021-00820-6.
4. An, X.; Li, W.; Lan, J.; Di, X.; Adnan, M. Seasonal co-pollution characteristics of parent-PAHs and alkylated-PAHs in karst mining area soil of Guizhou, Southwest China. *Frontiers in Environmental Science* **2022**, *10*, 990471, doi:10.3389/fenvs.2022.990471.
5. Niu, H.-y.; Sun, Q.-q.; Bu, Y.-c.; Chen, H.-y.; Yang, Y.-x.; Li, S.-p.; Sun, S.-w.; Mao, Z.-h.; Tao, M. Study of the microstructure and oxidation characteristics of residual coal in deep mines. *Journal of Cleaner Production* **2022**, *373*, 133923, doi:https://doi.org/10.1016/j.jclepro.2022.133923.
6. Li, W.; Zuo, Y.; Wang, L.; Wan, X.; Yang, J.; Liang, T.; Song, H.; Weihrauch, C.; Rinklebe, J. Abundance, spatial variation, and sources of rare earth elements in soils around ion-adsorbed rare earth mining areas. *Environmental Pollution* **2022**, *313*, 120099, doi:https://doi.org/10.1016/j.envpol.2022.120099.
7. Liu, S.; Yuan, R.; Wang, X.; Yan, Z. Soil tungsten contamination and health risk assessment of an abandoned tungsten mine site. *Science of The Total Environment* **2022**, *852*, 158461, doi:https://doi.org/10.1016/j.scitotenv.2022.158461.
8. Guo, J.; Xuan, F.; Li, D.; Wang, J.; Zhang, B. Variations of Soil Chemical Properties and Microbial Community around the Acid Reservoir in the Mining Area. *Sustainability* **2022**, *14*, doi:10.3390/su141710746.
9. Zheng, X.; Lu, A.; Gao, X.; Zhao, J.; Zheng, D. Contamination of heavy metals in soil present situation and method. *Soil and Environmental Sciences* **2002**.
10. Peng, H.; Chen, Y.; Li, J.; Lu, J. Energy information flow-based ecological risk transmission among

communities within the heavy metals contaminated soil system. *Chemosphere* **2022**, *287*, 132124, doi:https://doi.org/10.1016/j.chemosphere.2021.132124.
11. Qin, J.Q.; Xia, B.C.; Zhao, P.; Zhao, H.R.; Xie, G.H. Accumulation and Translocation of Cd in Two Miscanthus floridulus Populations. *Journal of Agro-Environment Science* **2011**.
12. QinJianqiao; Xiabeicheng; Peng, Z. Photosynthetic physiological response of two Miscanthus floridulus populations to Cd stress. *Acta Ecologica Sinica* **2010**, *20*, 288-299.
13. Wang, Y.; Ren, Z.; Ma, P.; Wang, Z.; Niu, D.; Fu, H.; Elser, J.J. Effects of grassland degradation on ecological stoichiometry of soil ecosystems on the Qinghai-Tibet Plateau. *Science of The Total Environment* **2020**, *722*, 137910, doi:https://doi.org/10.1016/j.scitotenv.2020.137910.
14. Liu, Y.; Yang, Y.; Li, C.; Ni, X.; Ma, W.; Wei, H. Assessing Soil Metal Levels in an Industrial Environment of Northwestern China and the Phytoremediation Potential of Its Native Plants. *Sustainability* **2018**, *10*, doi:10.3390/su10082686.
15. Qin, J.; Zhao, H.; Dai, M.; Zhao, P.; Chen, X.; Liu, H.; Lu, B. Speciation Distribution and Influencing Factors of Heavy Metals in Rhizosphere Soil of Miscanthus Floridulus in the Tailing Reservoir Area of Dabaoshan Iron Polymetallic Mine in Northern Guangdong. *Processes* **2022**, *10*, doi:10.3390/pr10061217.
16. Zhao, H.; Xia, B.; Fan, C.; Zhao, P.; Shen, S. Human health risk from soil heavy metal contamination under different land uses near Dabaoshan Mine, Southern China. *Sci Total Environ* **2012**, *417-418*, 45-54, doi:10.1016/j.scitotenv.2011.12.047.
17. Wu, Y.G.; Lin, C.; Tong, X.; Lu, W.; Zhu, L.; Chu, C.X.; Ling, Y.; Xu, S.J. Environmental impacts of acid mine drainage from the Dabaoshan Mine: Ⅱ. Agricultural ecosystem. **2005**.
18. Hongbin, W.; Wensheng, S.; Chongyu, L. Ecology for heavy metal pollution: recent advances and future prospects. *Acta ecologica snica* **2005**, *25*, 596-605.
19. Hsu, M.J.; Selvaraj, K.; Agoramoorthy, G. Taiwan's industrial heavy metal pollution threatens terrestrial biota. *Environmental Pollution* **2006**, *143*, 327-334.
20. Qin, J.Q.; Xia, B.C.; Meng, H.U.; Zhao, P.; Zhao, H.R.; Lin, X.F. Analysis of the Vegetation Succession of Tailing Wasteland of Dabaoshan Mine,Guangdong Province. *Journal of Agro-Environment Science* **2009**.
21. Lin, C.X.; Long, X.X.; Tong, X.L. Guangdong Dabaoshan mine: ecological degradation, acid drainage and possible measures for their remediation. *Ecologic Science* **2003**.
22. Wang, Y.; Dong, R.; Zhou, Y.; Luo, X. Characteristics of groundwater discharge to river and related heavy metal transportation in a mountain mining area of Dabaoshan, Southern China. *Sci Total Environ* **2019**, *679*, 346-358, doi:10.1016/j.scitotenv.2019.04.273.
23. Fan, L.; Zhao, W.; Feng, W.; Mo, P.; Zhao, Y.; Yang, G.; Xu, Z. Insight into the Characteristics of Soil Microbial Diversity during the Ecological Restoration of Mines: A Case Study in Dabaoshan Mining Area, China. *Sustainability* **2021**, *13*, doi:10.3390/su132111684.
24. Zhao, H.; Xia, B.; Qin, J.; Zhang, J. Hydrogeochemical and mineralogical characteristics related to heavy metal attenuation in a stream polluted by acid mine drainage: A case study in Dabaoshan Mine, China. *Journal of Environmental Sciences* **2012**, *24*, 979-989, doi:10.1016/s1001-0742(11)60868-1.
25. Yang, C.; Lu, G.; Chen, M.; Xie, Y.; Guo, C.; Reinfelder, J.; Yi, X.; Wang, H.; Dang, Z. Spatial and temporal distributions of sulfur species in paddy soils affected by acid mine drainage in Dabaoshan sulfide mining area, South China. *Geoderma* **2016**, *281*, 21-29, doi:10.1016/j.geoderma.2016.06.032.
26. Zhou, J.M.; Dang, Z.; Situ, Y. Distribution and characteristics of heavy metals contaminations in soils from Dabaoshan Mine Area. *Journal of Agro-environmental Science* **2004**.
27. Sun, Q. Chemical Properties of Lead-Zinc Tailings at Disposal Ground. *Rural Eco-environment* **2000**.

28. Shanming, F.U.; Yongzhang, Z.; Yuyan, Z.; Feng, Z.; Quanzhou, G.; Xianzhi, P.; Zhi, D.; Chengbo, Z.; Xiaoqiang, Y.; Zhijun, Y. Study on Heavy Metals in Soils Contaminated by Acid Mine Drainage from Dabaoshan Mine, Guangdong. *Environment Science* **2007**, *28*, 805-812.
29. Lu, K. Analytical Methods of Soil and Agricultural Chemistry. **1999**.
30. Guan, S.Y.; Zhang, D.; Zhang, Z. Soil enzyme and its research methods. **1986**.
31. Stanton, D.E.; Batterman, S.A.; Von Fischer, J.C.; Hedin, L.O. Rapid nitrogen fixation by canopy microbiome in tropical forest determined by both phosphorus and molybdenum. *Ecology* **2019**, *100*, e02795, doi:https://doi.org/10.1002/ecy.2795.
32. Geisseler, D.; Horwath, W.R. Investigating amino acid utilization by soil microorganisms using compound specific stable isotope analysis. *Soil Biology and Biochemistry* **2014**, *74*, 100-105, doi:https://doi.org/10.1016/j.soilbio.2014.02.024.
33. Vance, E. An extraction method for measuring soil microbial biomass C. *Soil Biol Biochem* **1987**, *19*.
34. Ge, Y.; Wang, Q.; Wang, L.; Liu, W.; Christie, P. Response of soil enzymes and microbial communities to root extracts of the alien Alternanthera philoxeroides. *Archives of Agronomy and Soil Science* **2017**, *64*.
35. Korona-Glowniak, I.G.-L.A.L.A.B.T.M.A.T.I.V.A.o.E.O.a.H.P.G.; Urease, A. *Molecules* **2020**, *25*, doi:10.3390/molecules25030586.
36. Quastel, J.H. Urease Activity and Ascorbic Acid. *Nature* **1943**, *152*, 215-215, doi:10.1038/152215b0.
37. Li, J.; Wei, Y.-Y.; Xu, Z.-R. Visual detection of acid phosphatase based on hollow mesoporous manganese dioxide nanospheres. *Analytica Chimica Acta* **2020**, *1138*, 1-8, doi:https://doi.org/10.1016/j.aca.2020.09.010.
38. Shan, Q.; Yu, Y.; Yu, J.; Zhang, J. Soil enzyme activities and their indication for fertility of urban forest soil. *Frontiers of Environmental Science & Engineering in China* **2008**, *2*, 218-223, doi:10.1007/s11783-008-0037-5.
39. Sun, Y.; Liu, H.; Tan, X.; Li, Z.; Du, Y.; Zheng, A.; Liu, X.; Peng, N. Highly efficient redox reaction between potassium permanganate and 3,3′,5,5′-tetramethylbenzidine for application in hydrogen peroxide based colorimetric assays. *RSC Advances* **2019**, *9*, 1889-1894, doi:10.1039/C8RA07758D.
40. Xu, H.; Brookes, J.; Hobson, P.; Pei, H. Impact of copper sulphate, potassium permanganate, and hydrogen peroxide on Pseudanabaena galeata cell integrity, release and degradation of 2-methylisoborneol. *Water Research* **2019**, *157*, 64-73, doi:https://doi.org/10.1016/j.watres.2019.03.082.
41. Chalabi, M.; Khademi, F.; Yarani, R.; Mostafaie, A. Proteolytic Activities of Kiwifruit Actinidin (Actinidia deliciosa cv. Hayward) on Different Fibrous and Globular Proteins: A Comparative Study of Actinidin with Papain. *Applied Biochemistry and Biotechnology* **2014**, *172*, 4025-4037, doi:10.1007/s12010-014-0812-7.
42. Bandyopadhyay, S.; Maiti, S.K. Different Soil Factors Influencing Dehydrogenase Activity in Mine Degraded Lands—State-of-Art Review. *Water, Air, & Soil Pollution* **2021**, *232*, 360, doi:10.1007/s11270-021-05302-0.
43. Nannipieri, P. The potential use of soil enzymes as indicators of productivity, sustainability and pollution. *Grace* **1994**, 238-244.
44. Wu, B.; Luo, S.; Luo, H.; Huang, H.; Xu, F.; Feng, S.; Xu, H. Improved phytoremediation of heavy metal contaminated soils by Miscanthus floridulus under a varied rhizosphere ecological characteristic. *Sci Total Environ* **2022**, *808*, 151995, doi:10.1016/j.scitotenv.2021.151995.
45. Yu, H.; Zheng, X.; Weng, W.; Yan, X.; Chen, P.; Liu, X.; Peng, T.; Zhong, Q.; Xu, K.; Wang, C.; et al. Synergistic effects of antimony and arsenic contaminations on bacterial, archaeal and fungal

communities in the rhizosphere of Miscanthus sinensis: Insights for nitrification and carbon mineralization. *J Hazard Mater* **2021**, *411*, 125094, doi:10.1016/j.jhazmat.2021.125094.
46. Insam, H.; Hutchinson, T.C.; Reber, H.H. Effects of heavy metal stress on the metabolic quotient of the soil microflora. *Soil Biology & Biochemistry* **1996**, *28*, 691-694.
47. Zhuang, P.; McBride, M.B.; Xia, H.; Li, N.; Li, Z. Health risk from heavy metals via consumption of food crops in the vicinity of Dabaoshan mine, South China. *Sci Total Environ* **2009**, *407*, 1551-1561, doi:10.1016/j.scitotenv.2008.10.061.
48. Chander, K.; Brookes, P.C. Microbial biomass dynamics during the decomposition of glucose and maize in metal-contaminated and non-contaminated soils. *Soil Biology & Biochemistry* **1991**, *23*, 917-925.
49. R., D.; Bardgett; and; S.; Saggar. Effects of heavy metal contamination on the short-term decomposition of labelled [14C]glucose in a pasture soil. *Soil Biology & Biochemistry* **1994**.
50. Chen, M.; Lu, G.; Guo, C.; Yang, C.; Wu, J.; Huang, W.; Yee, N.; Dang, Z. Sulfate migration in a river affected by acid mine drainage from the Dabaoshan mining area, South China. *Chemosphere* **2015**, *119*, 734-743, doi:10.1016/j.chemosphere.2014.07.094.
51. Jiang-Tao, B.I.; Da-Han, H.E.; Huang, Z.Y. Response of Soil Microbial Population Number and Distribution to Vegetation Restoration in Degraded Ecological System. *Scientia Geographica Sinica* **2009**.
52. Bao, Y.; Guo, C.; Lu, G.; Yi, X.; Wang, H.; Dang, Z. Role of microbial activity in Fe(III) hydroxysulfate mineral transformations in an acid mine drainage-impacted site from the Dabaoshan Mine. *Sci Total Environ* **2018**, *616-617*, 647-657, doi:10.1016/j.scitotenv.2017.10.273.
53. Liu, Q.; Chen, B.; Haderlein, S.; Gopalakrishnan, G.; Zhou, Y. Characteristics and environmental response of secondary minerals in AMD from Dabaoshan Mine, South China. *Ecotoxicol Environ Saf* **2018**, *155*, 50-58, doi:10.1016/j.ecoenv.2018.02.017.
54. He, W.X.; Zhu, M.E.; Zhang, Y.P. Recent advance in relationship between soil enzymes and heavy metals. *Soil and Environmental Sciences* **2000**.
55. Wang, X.; Li, L.; Guo, N.; Xin, Z.; Li, X.; Sun, X.; Li, Y. A Comprehensive Exploration on Pollution Characteristics and Ecological Risks of Heavy Metals in Surface Paddy Soils around a Large Copper Smelter, Southeast China. *Sustainability* **2021**, *13*, doi:10.3390/su132313359.
56. He, W.X.; Chen, H.M.; Feng, G.Y. Study on enzyme index in soils polluted by mercury, chromium and arsenic. *ACTA ENTIAE CIRCUMSTANTIAE* **2000**.
57. Yang, Z.X.; Liu, S.Q. Influence of compound pollution of heavy metals on rape. *Journal of Agricultural University of Hebei* **2000**, 27-30.
58. Muller, G. Index of Geoaccumulation in Sediments of the Rhine River. *GeoJournal* **1969**, *2*, 109-118.
59. Heling, D.; Rothe, P.; F?Rstner, U.; Stoffers, P. Sediments and Environmental Geochemistry || Sediment Criteria Development. **1990**, *10.1007/978-3-642-75097-7*, 311-338.
60. Ying, T.; Huang, C.; Jian, L.; Yao, H.; Liu, F. Enzyme activities in soils contaminated by abandoned copper tailings. *The journal of applied ecology* **2003**, *14*, 1976-1980.

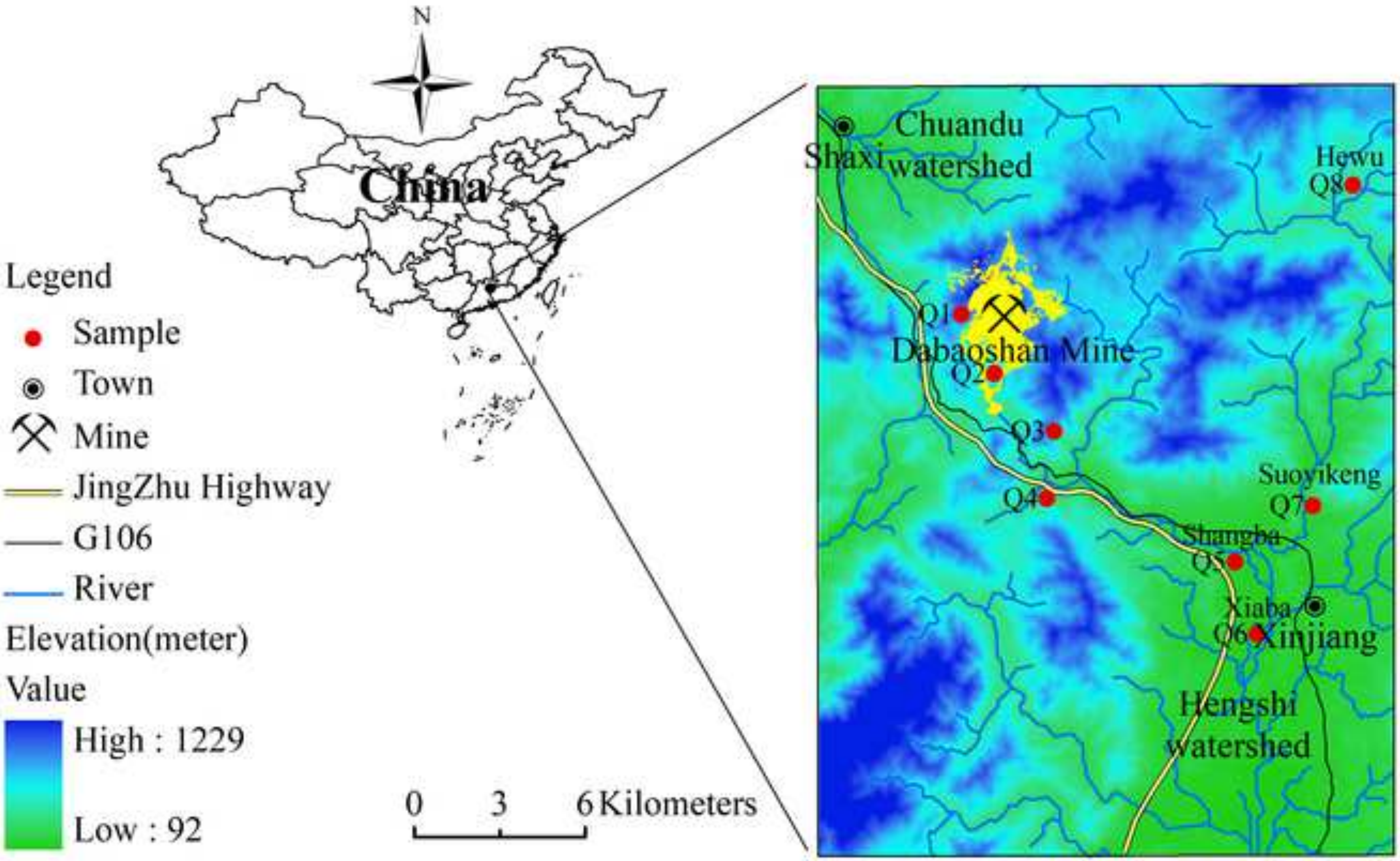
N
China
Legend
Sample
Town
Mine
JingZhu Highway
G106
River
Elevation(meter)
Value
High : 1229
Low : 92
0 3 6 Kilometers
Chuandu watershed
Shaxi
Hewu
Q8
Q1
Dabaoshan Mine
Q2
Q3
Q4
Suoyikeng
Q7
Shangba
Q5
Xiaba
Q6
Xinjiang
Hengshi watershed

Click here to access/download;Figure;Fig 2.tif

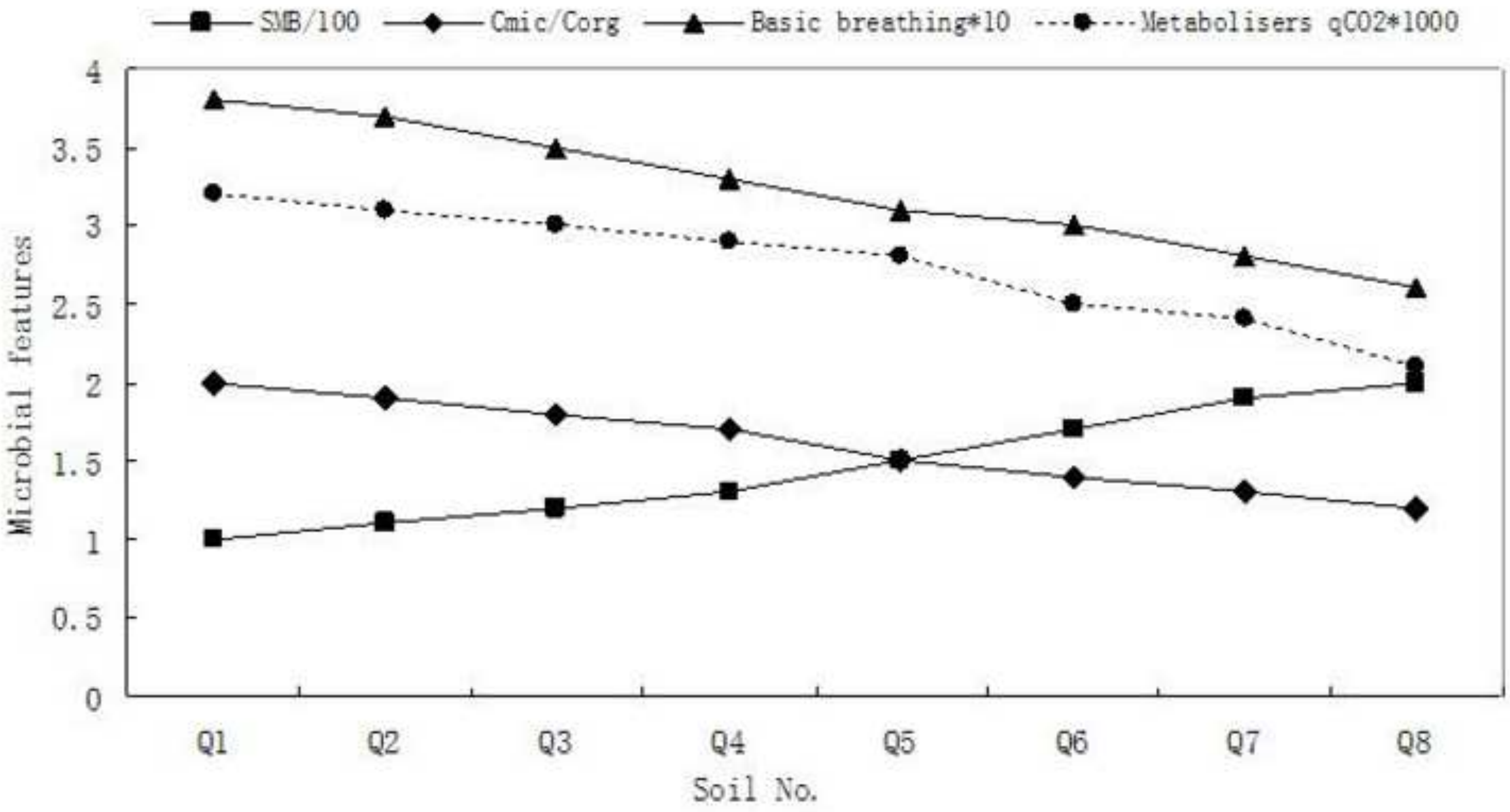

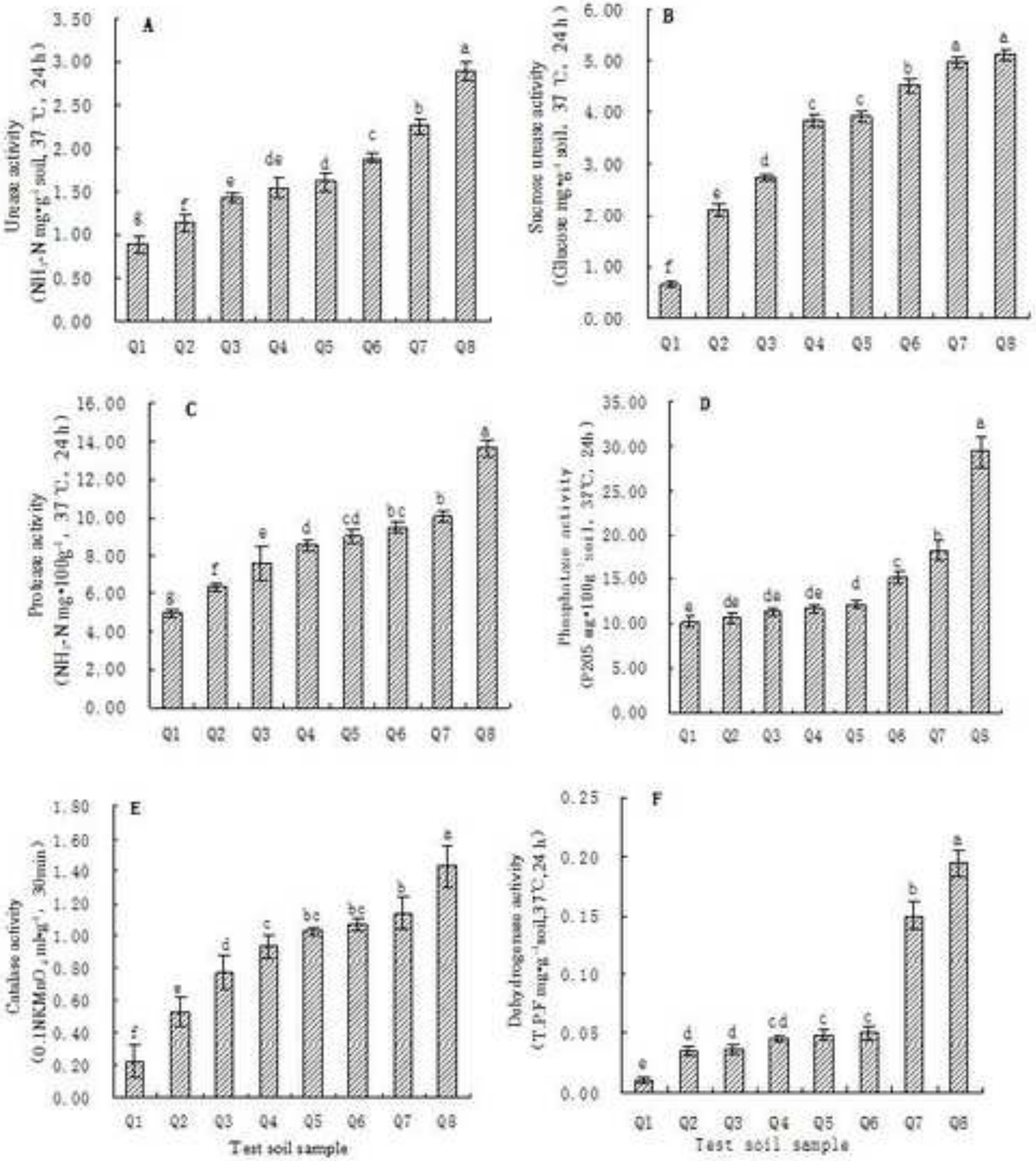

Fig.3 The enzymatic activities of soil samples tested ((A) is the Urease activity,(B) is the Sucrose enzyme activity, (C) is the Protease activity, (D) is the Phosphatase activity, (E) is the Catalase activity,and (F) is the Dehydrogenase activity.)

Note: Error bars mean standard deviation; different letters in the same group indicate significant difference at $P<0.05$ according to Duncan's multiple range tests.
