# Supplementary material for: Heavy metal content and microbial characteristics of soil plant system in Dabaoshan mining area, Guangdong Province
Source: PLoS One. 2023 Jun 9;18(6):e0285425. doi: 10.1371/journal.pone.0285425 (PMC10256142; doi:10.1371/journal.pone.0285425)
Supplement: Attachment — Submitted filename: Review report PONE-D-22-31232.docx [file pone.0285425.s003.docx]

**Review Report**

The manuscript entitled **“Microbial characteristics of soil-Miscanthus floridulus plant system in Dabaoshan Leadzinc mining area, northern Guangdong Province”** is interesting. However, I have added some corrections in the PDF manuscript file and suggest the following points that may help to improve the work:

Please rewrite title in more simple and effective way.

The introduction should be extended to discuss related literature, hypothesis and research aim in details.

Please explain the basis of using Miscanthus floridulus as test plant species.

The discussion section still needs improvement, and should be linked to the findings of the previous reports on this topic.

Conclusion section should be concise with future direction of research work at the end.
