# Supplementary material for: Heavy metal content and microbial characteristics of soil plant system in Dabaoshan mining area, Guangdong Province
Source: PLoS One. 2023 Jun 9;18(6):e0285425. doi: 10.1371/journal.pone.0285425 (PMC10256142; doi:10.1371/journal.pone.0285425)
Supplement: Attachment — Submitted filename: Response to Reviewers.docx [file pone.0285425.s004.docx]

Response to Reviewer 1 Comments

**Point 1:** Please rewrite title in more simple and effective way.

**Response 1:** Thank you for your valuable comments. We have changed the title of the paper to: “Heavy metal content and microbial characteristics of soil plant system in Dabaoshan mining area, Guangdong Province”.

**Point 2:** The introduction should be extended to discuss related literature, hypothesis and research aim in details.

**Response 2:** Thank you for your advice. The relevant literature, hypotheses and research objectives are further discussed in the introduction.

**Point 3:** Please explain the basis of using Miscanthus floridulus as test plant species.

**Response 3:** Thank you very much for your constructive comments. Here is an explanation added to the text,

“Miscanthus floridulus is a perennial herb plant of the Miscanthus, which is widely distributed in southern China and has strong adaptability [14,15]. It is an ideal plant for phytoremediation due to its rapid growth, large biomass and well-developed root system, which can accumulate heavy metals and reduce their mobility and availability [16,17].”

**Point 4:** The discussion section still needs improvement, and should be linked to the findings of the previous reports on this topic.

**Response 4:** Thank you very much for your constructive comments. The results of the experiment and the discussion part were done together; This time, the discussion section has been strengthened based on the test results.

**Point 5:** Conclusion section should be concise with future direction of research work at the end.

**Response 5:** Thank you very much for your constructive comments. The conclusions have been reviewed and revised to add directions for future work. Please look at the revised paper, thank you.

Response to Reviewer 2 Comments

**Point 1:** Title seems to be incomplete since the microbial characteristics were not the only set of parameters studied by the authors in this study.

**Response 1:** Thank you for your comments. The title of the paper has been revised to " Heavy metal content and microbial characteristics of soil plant system in Dabaoshan mining area, Guangdong Province".

**Point 2:** The abstract lacking the objective of the study. The authors need to state what is the rational of this study in the abstract

**Response 2:** Thank you for your advice. Here the abstract is reviewed and modified, and the purpose of the study is added.

**Point 3:** I don’t know why there’s no line and page numbering in the ms making things difficult for reviewer to spot and convey the mistakes

**Response 3:** Thank you for your advice. Here the paper has been checked and modified to make it more standardized.

**Point 4:** Introduction Line 1: South of the China

**Response 4:** Thank you for your suggestion. The problem has been solved. The first sentence has been modified here, please see the modification trace, thank you.

**Point 5:** Introduction is too short and faulty. The authors focused a plant species Miscanthus floridulus in the title and mentioned nothing about this plant in the introduction. Moreover, authors may also introduce the readers about Dabaoshan Lead-zinc mining area in the introduction section

**Response 5:** Thank you for your advice. Here, in the introduction part, the introduction of the research on Miscanthus floridulus is added. In addition, the introduction of Dabaoshan lead-zinc mining area is added in the introduction.

**Point 6:** The objective and hypothesis of the study at the end of the introduction is all over the place e.g., the authors in the current never made an attempt to collect super accumulated plant germplasm resources. Authors need to focus on what they have experimented only

**Response 6:** Thank you for your advice. Here, the research objectives and assumptions at the end of the introduction are reasonably revised to make them more close to the actual situation of this study. Thank you.

**Point 7:** Too long introduction of the study area in M&M section. This may be summarized here and introductory sentences may be shifted to introduction

**Response 7:** Thank you for your comments. Here, the introduction of the study area is simplified.

**Point 8:** “Miscanthus floridulus community was dominant, accounting for more than 70%, with 60% ~ 80% coverage” This sentence is without any reference. Again authors are putting extra information in M&M some of which may be shifted to Introduction with references

**Response 8:** Thank you for your comments. References have been added here, please refer to the revised paper for details.

**Point 9:** In the ms, Miscanthus floridulus is written without italicized at many places. Please take care

**Response 9:** Thank you for your advice. Here, the corresponding phrase is italicized and checked. Thanks a lot.

**Point 10:** In M&M some instruments e.g., Atomic Absorption Spectrophotometer is mentioned without make and model

**Response 10:** Thank you for your advice. This time, the manufacturer and model of the atomic absorption spectrophotometer are given here.

**Point 11:** Why the authors measured Zn, Pb, Cu, Cd with ICP-OES, why not the other heavy metals too?

**Response 11:** Thank you for your advice. Here, according to the previous research results, the most polluted heavy metals in Dabaoshan mining area are "zinc, lead, copper and cadmium", so only these four heavy metals have been measured.

**Point 12:** 3.1.3 M. floridulus on the relationship between mango plants and soil heavy metal elements……I am unable to understand what is mango here?

**Response 12:** Thank you for your advice. Here is a clerical error. The sentence has been revised as follows: Relationship between M. floridulus and soil heavy metals

**Point 13:** The legends of the figures are embedded in the text without the figures in place; however they are also presented at the bottom with figures in place. I think remove them from the text

**Response13:** Here, the corresponding questions have been modified.

Point 14: I feel that the authors presented the results strongly but discussion is partly weak. Please take care

**Response 14:** Thank you for your advice. This is revised. Please see the revised paper for details.

**Point 15:** Why there is no statistical analysis in table 3 and 4?

**Response 15:** Thank you for your advice. Because the difference is not obvious and is not the focus of the study, there is no difference test.

**Point 16:** I am not expert in English language; however, I spotted some typographical and syntax errors in the ms which authors can double check

**Response 16:** Thank you for your advice. This is revised. Please see the revised paper for details.
